# Navigating regulatory challenges, technical performance and circular economy integration of mineral-based waste materials for sustainable construction: A mini review in the European context

**DOI:** 10.1177/0734242X241270973

**Published:** 2024-09-05

**Authors:** Samuel J. Armistead, Arezou Babaahmadi

**Affiliations:** Department of Architecture & Civil Engineering, Chalmers University of Technology, Gothenburg, Sweden

**Keywords:** Waste utilization, mineral-based waste, supplementary cementitious materials, circular and sustainable construction, regulatory barriers analysis

## Abstract

The integration of mineral-based waste materials is crucial for achieving a sustainable and circular construction sector. Whilst technological and economic aspects receive attention, this mini review spotlights overlooked legal ‘regulatory hurdles’. It explores major barriers within the European Union, aiming to compress the current ~30-year material development pipeline. Significant hurdles include the absence of harmonized end-of-waste criteria (Waste Framework Directive), the need for consensus-building in chemical risk assessments (REACH & CLP), scarcity of up-to-date harmonized product standards (Construction Products Regulation) and precision values for limit state analysis in structural codes (Eurocodes). This mini review serves as a practical manual, outlining the intricate regulatory landscape for industry experts, regulators and researchers. Emphasizing the parallel importance of environmental safety considerations and performance, our study presented in this mini-review, underscores the necessity for a multi-stakeholder approach to alleviate regulatory barriers. By illuminating regulatory intricacies, this mini review establishes the foundations for wider discussions and in-depth analysis as to the future outlook for consensus development procedures in a rapidly changing and challenging global construction sector. The manuscript also provides stakeholders with vital insights for informed decision-making, helping to facilitate the paradigm shift towards a sustainable and circular construction sector.

## Introduction

The cement industry stands as a major contributor to anthropogenic CO_2_ production, constituting approximately 8% of total emissions (Olsson et al., 2023). In response to this environmental challenge, the utilization of industrial by-products in the cement industry has emerged as a key strategy for decarbonization over several decades (Siddique, 2014). Aligned with the Global Cement and Concrete Associations roadmap to achieve net-zero emissions by 2050, significant savings in clinker, cement and binder production, constituting 20% of the overall 3.8 Gt strategy, have been outlined (Global Cement and Concrete Association, 2021). This approach not only contributes to decarbonization but also aligns with the principles of the circular economy, offering additional benefits in terms of resource efficiency and waste reduction (Marsh et al., 2022).

Despite these positive strides towards sustainability, concerns arise regarding the scarcity of common supplementary cementitious materials (SCMs) necessary for these initiatives. In pursuit of climate objectives, over 20% of Europe’s coal combustion plants have closed in the last 5 years, drastically reducing the availability of fly ash, a pivotal component accounting for a third of the SCM market (Horváth et al., 2022; Scrivener, 2014). Simultaneously, a contracting European steel industry has raised the price and limited the availability of commonly used SCM, ground granulated blast furnace slag (Skoczkowski et al., 2020). This scarcity has fuelled a growing interest among researchers and industrial entities to explore new markets producing industrial by-products suitable as alternative cements, securing the supply chain necessary for a sustainable construction sector (Habert et al., 2020; Lehne and Preston, 2018).

An SCM, defined as a material displaying hydraulic or pozzolanic activity, is central to these sustainability endeavours. Pozzolans, containing high silica/alumina contents, react with portlandite minerals produced during cement hydration, creating additional cementitious matrices (He et al., 1995). This diverse category includes natural pozzolans primarily derived from volcanic sources, sedimentary-based diatomic earth, naturally burned clay-based pozzolans and artificial pozzolans sourced from mineral-based industrial wastes (Snellings et al., 2012).

Despite the significant growth in research related to SCM utilization in the construction sector (18% growth rate compared to the average growth rate across all scientific fields at 4%–5%), with over 25,000 articles published from 2017 to 2022 (Snellings et al., 2023), a major concern looms. Materials with extensive investigations and documented technical potentials as cement replacements have yet to find substantial applications in real-world scenarios. With impending supply chain risks and the urgent need to shift towards a circular and sustainable construction sector, it becomes key to identify barriers impeding the widespread utilization of industrial waste as SCMs.

Although economic and technical drivers and barriers to utilizing mineral-based waste-derived SCMs have been explored (Scrivener et al., 2018), little focus has been directed towards the legal and regulatory-based hurdles critical for these materials to access the European construction market (Dewald and Achternbosch, 2016). These regulations cover materials as they transition from being a waste to their secondary material chemical status, to being used as a product within structures. Supported by a well-established standardization community, these regulations aim to produce consensus-based state-of-the-art specifications, ensuring safety throughout the European Union (EU). However, this conservative approach is at odds with the demands industry faces in the face of impending digital and sustainable revolutions. It is critical to explore these consensus-based systems for their barriers to reduce the current ~30-year timeline for widespread acceptance and meet climate objectives (Scrivener, 2014).

Within this article, we delve into the regulatory journey of mineral-based waste-derived SCMs, examining their path from production to application. The EU has been chosen as it represents a global leader in consensus-based market systems and associated development procedures. Analysing the formal processes associated with each regulatory act, we aim to identify and highlight barriers hindering the widespread adoption of new SCMs. The purpose of this article is to emphasize the importance of awareness regarding parameters affecting the transition of these products from the lab to the field. Although engaging in this discussion, it is crucial to clarify our intention is not to criticize environmental laws, but rather to foster a constructive dialogue and explore potential areas for improvement.

In tandem with this exploration, we recognize that technical performance is a crucial facet of importance in the recycling SCMs. As we delve into the regulatory journey of mineral-based waste-derived SCMs within the EU, we emphasize that technical performance must be considered hand in hand with environmental laws. This integrated approach ensures that the adoption of SCMs not only aligns with sustainability goals but also meets the necessary performance benchmarks in real-world applications.

Moreover, within the current trend of focusing on cement replacement, translating performance as reactivity, there is a need to broaden the discussion. In the context of circular economy principles, recycling mineral-based waste, even as aggregate or inert filler, becomes a meaningful consideration. Understanding performance in these diverse contexts and discerning between them is pivotal. This distinction is often a point of misunderstanding for experts and industrial bodies striving to explore recycling options for their produced waste. Therefore, this article aims to shed light on the multifaceted nature of performance evaluation, essential for progressing sustainable construction practices and circular economy principles.

## Methodology

Our methodology aligns with the ethos of interdisciplinary environmental research, synergizing diverse resources to shape a discussion forum that serves as a manual for researchers, industrial bodies, law makers and stakeholders in this area. Leveraging existing literature, regulatory documents and expert insights, we meticulously craft a comprehensive panorama of the regulatory journey of mineral-based waste-derived construction materials within the EU. This inclusive approach allows us to dissect the formal intricacies associated with regulatory acts, pinpointing barriers that impede the seamless transition of these materials from the laboratory to tangible applications. Our synthesis of insights from varied sources aspires to provide a nuanced perspective, fostering informed discussions on the regulatory challenges surrounding SCMs in the construction sector. In unveiling these barriers, we aim to contribute to a clearer understanding of the regulatory landscape, facilitating the sustainable and effective integration mineral-based waste as new SCMs, possible recycled aggregate or filler materials into mainstream construction practices.

## Holistic regulatory landscape

The pursuit of sustainable construction materials involves navigating a complex regulatory landscape, necessitating an understanding of both technical and legal dimensions. In the EU, the Waste Framework Directive plays a pivotal role, defining waste and by-products. Mineral-based waste-derived SCMs for application in construction fall into a unique category – while initially waste, their reclassification as by-products hinges on meeting construction sector needs and stringent environmental criteria. This intersection of waste and by-products illustrates a key challenge and opportunity within the regulatory framework.

### EU waste directive

According to Directive (2008/98/EC) of the European Parliament and of the council on waste and repealing certain directives, ‘waste’ means any substance or object which the holder discards or intends or is required to discard (*Official Journal of the European Union*, 2008b). By-products, however, are substances or objects resulting from a production process, the primary aim of which is not the production of that substance, or object, is considered not to be waste, whilst also fulfilling several conditions (Table 1). Therefore, mineral-based waste-derived SCMs, whilst naturally waste, if proven to be both beneficial for the construction sector and able to cope with product, environmental and health protection requirements, would be re-classified as a by-product.

**Table 1.** Required criteria to fulfil to be classified as a by-product (*Official Journal of the European Union*, 2008b).

Classification of by-product
(A) Further use of the substance or object is certain.
(B) The substance or object can be used directly without any further processing other than normal industrial practice.
(C) The substance or object is produced as an integral part of a production process.
(D) Further use is lawful, that is, the substance or object fulfils all relevant product, environmental and health protection requirements for the specific use and will not lead to overall adverse environmental or human health impacts.

The waste directive also considers the steps towards preventing waste, the primary aim of the waste management hierarchy. For several waste types, there are end-of-waste criteria, meaning that waste has undergone recycling or other recovery operation, resulting in a ‘secondary material’ that no longer exhibits the characteristics of waste. End-of-waste status can only be reached if the material complies with a number of conditions (Table 2).

**Table 2.** Required criteria to transfer from waste to secondary material classification (*Official Journal of the European Union*, 2008b).

Classification of end-of-waste
(A) The substance or object is to be used for specific purposes.
(B) A market or demand exists for such a substance or object.
(C) The substance or object fulfils the technical requirements for the specific purposes and meets the existing legislation and standards applicable to products.
(D) The use of the substance or object will not lead to overall adverse environmental or human health impacts: (i) Without risk to water, air, soil, plants or animals. (ii) Without causing a nuisance through noise or odours. (iii) Without adversely affecting the countryside or places of special interest.

For some waste types (scrap metal, glass), EU-wide end-of-waste criteria have been established (Council of the European Union, 2011; *Official Journal of the European Union*, 2012). Within these documents, the criteria to be fulfilled in terms of technical performance and levels of potentially hazardous materials and substances, for the waste to be counted as recycled as secondary material, is clearly described. This clarity provides greater security and shorter lead times, as an official decision in each individual case is not required.

### Product regulations

Chemical, product and structural regulations all apply when considering a primary material used within construction. Here, we therefore assess the barriers in applying each regulation for a mineral-based waste-derived SCMs.

#### REACH & CLP

When producing a material in quantities above 1 tonne, it is required to be registered with the Registration, Evaluation, Authorization and Restriction of Chemicals regulation, also known as REACH, which aims to protect the environment and human health by requiring that all chemical substances are assessed for their risk (*Official Journal of the European Union*, 2006). As opposed to EoW criteria, which are administered by national authorities, the REACH regulation is managed by the European Chemicals Agency (ECHA), which has the authority to ban substances if their risks are ‘unmanageable’. REACH ensures that manufacturers are responsible for the safe use of substances, typically through the production of a safety data sheet, when introduced to the market (European Chemicals Agency, 2007).

In tandem with the REACH regulation, the classification, labelling and packaging regulation, also known as the CLP regulation, is key for communicating the potential hazards associated with a substance or mixture (*Official Journal of the European Union*, 2008a). The CLP sets detailed physical, health and environmental criteria for classifying and labelling, allowing for risk management through the substance and mixture value chain. The CLP requires each manufacturer to notify the ECHA C&L inventory as to any substance placed on the market. The regulation is based on the United Nations, Globally Harmonized System that is designed to ensure a high level of protection of health and the environment (European Chemicals Agency, 2015).

**Figure 1.**
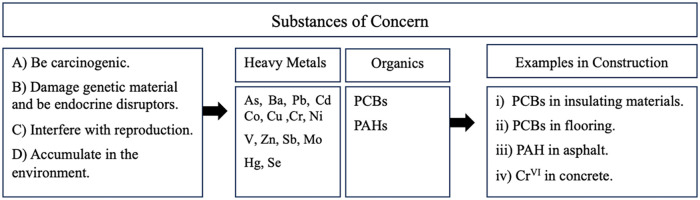
Schematic summarizing the criteria, relevant chemical constituents and examples of use within construction for substances of concern.

If a substance is known to be a hazard to human health or the environment, fulfilling a set of criteria (Figure 1) and is contained in the produced material in more than 0.1% by weight, the ECHA must be notified. There are many examples of potential sources of these substances within construction wastes (Figure 1) (Hjelmar et al., 2016).

##### Construction Products Regulation

Once REACH & CLP registered, in order to be used within a construction product, an SCM must comply with the Construction Products Regulation (CPR) (No 305/2011) (*Official Journal of the European Union*, 2011). The CPR outlines (Annex I) the essential characteristics (Table 3) required to be marketed as a construction product with the European market (European Commission, 2022a). Instead of outlining prescribed requirement thresholds for products, the CPR outlines the steps required to formally assess and communicate the performance of a material (Publications Office of the European Union, 2022). This is achieved through a document known as a ‘declaration of performance’, which is communicated via Conformite Europeenne (CE) marking on the product. In displaying CE marking, the manufacturer is stating that the product ‘conforms’ and has been appropriately assessed to the stated declaration of performance, which covers the essential requirements deemed to be relevant for the product use, and therefore allows for free circulation of the material throughout the single market (European Commission, 2015).

**Table 3.** Essential requirements for a construction product to achieve market access in the European Union (*Official Journal of the European Union*, 2011).

Construction products regulation: essential requirements
(i) Mechanical resistance and stability.
(ii) Safety in case of fire.
(iii) Hygiene health and the environment.
(iv) Safety and accessibility in use.
(v) Protection against noise.
(vi) Energy economy and heat retention.
(vii) Sustainable use of natural resource.

As with REACH regulations, the manufacturer is responsible for carrying out a risk analysis for the product, in the context of the essential requirements outlined in CPR. There are then multiple routes for establishing that the product conforms to these essential requirements. The first is through harmonized product standards, which have been published in the *Official Journal of the European Union* (OJEU). Harmonized standardization product standards are developed through the European Standardization Organizations, after receiving a mandate from the European Commission. A second route, originally designed for more innovative construction products, is through product-specific European Technical Assessments, also published in the OJEU. Within both these avenues, the consensus of interested parties throughout Europe, on a technical solution, is seen to reflect the state of the art for a given essential requirement, and through publication in the OJEU is accepted by authorities to create the possibility for ‘presumption of conformity’. Presumption of conformity is a benefit to economic operators, which does not describe performance, but allows for free circulation of products within the EU if they comply with the harmonized technical documents (Colombo and Eliantonio, 2017). Compliance is typically assessed by third-party technical assessment bodies, also known as TABs, which have received accreditation to perform a particular assessment. At the member state level, type testing schemes are typically available to show that a product meets national building regulatory requirements; however, only when no route to CE marking is possible (Research Institute of Sweden, 2023).

##### Eurocodes

When using a construction product within a structural capacity, in order to gain the benefit of ‘presumption of conformity’ with essential requirements: mechanical resistance and stability and safety in case of fire, it must be designed using Eurocode technical specifications (Gulvanessian et al., 2012). The Eurocodes are a series of 10 standards (EN 1990–EN 1999) produced by CEN /TC 250, which provide a comprehensive and common approach for designing products and engineering solutions for the built environment (European Commission, 2023). The standards provide design calculations for products playing a structural role in construction works (*Official Journal of the European Union*, 2003). Typically, characteristic values outlined in product technical specifications (harmonized standards/technical assessments) are integrated into Eurocode design calculations to determine the ultimate performance of a product in situ, also known as the design values (Gulvanessian et al., 2012). Characteristics values outlined within product standards are typically determined via the testing (at least 30 times), in a controlled manner (procedure, environment), achieving a statistical confidence of the materials characteristic (5% fractile) (Vu et al., 2020). Design values are then either calculated statistically (0.01% fractile) or calculated through applying ‘partial safety factors’ to ensure essential mechanical requirements are met (Gulvanessian et al., 2012). Partial factors also include national determined parameters, which are set by member states and serve to ensure an adequate level of safety is achieved at the national, regional and local levels, factoring in, when necessary, considerations such as climate and geology (Sousa et al., 2019). The ‘limit state concept’ is then applied to ensure that no potential loadings or actions result in the failure of the structure through its designated lifetime.

When designing a structure for its lifetime, also known as its service life, it is key that potential deterioration pathways are known, and therefore the appropriate use of a material within a project is clear. For example, within concrete structures: carbonation, chloride ingress, frost, alkali-silica interactions and general/associated cracking all contribute to the deterioration of structures, and therefore, a reduced strength over time. These effects are typically predicted from the underlying physics, through empirical modelling, or more recently through thermodynamic-based models. It is key that these frameworks are translated to new materials and formulations to ensure that a limit state is not reached during its service life.

## Insights and challenges in the recycling regulation for the cement and construction industry

Transitioning from a broad exploration of general product regulations to a focused examination on recycling within the cement and construction industry raises critical questions. Firstly, in the context of recycling waste for application in cement and construction, a key consideration is whether products can be defined as safe for use, when categorized as waste. Furthermore, determining the pertinent performance criteria becomes pivotal. The following discussion delves into these complex issues, providing insights and addressing challenges in recycling regulations for the cement and construction sector.

### Environmental safety regulations for mineral-based waste

The first important question to answer is whether there is any end of waste criteria for mineral-based waste-derived materials to be used as an SCM, aggregate or filler. Today, however, there are no specific end-of-waste criteria (at the EU level) for these purposes. Consequently, it is rather confusing for researchers or industrial bodies to judge how hazardous their mineral waste is and whether it has the potential to be used as a secondary material. In Figure 1, a schematic is provided summarizing the criteria, relevant chemical constituents and examples of use within construction for substances of concern.

**Figure 2.**
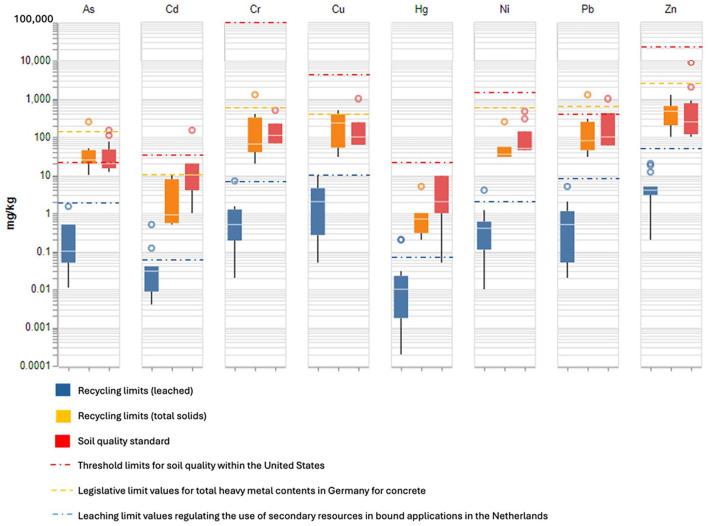
National standards (Australia, Belgium, Bulgaria, Czech, Denmark, Finland, France, Germany, Japan, Republic of Korea, Netherlands, Norway, Poland, Russia, Sweden, Switzerland, Thailand and UK) on soil environmental quality based on Provoost et al. (2006), as well as national (Austria, Belgium, Czech Republic, Denmark, Finland, France, Germany and Sweden) recycling limits (total solids), as well as leaching limits based on Joint Research Centre et al. (2012, 2014). Individual legislative limit values (United States, Germany and Netherlands) have been plotted for comparison (Provoost et al., 2006; Snellings et al., 2023). Outliners are presented as point data.

For currently used SCMs (fly ash, slag), many national, as opposed to European, end-of-waste frameworks enforce limit values for chemical constituents that may present environmental or human health risks, for a particular intended application or product. These values, however, are not easily accessible and often vary between member states. When considering mineral-based waste, a primary environmental and human health concern is the potential for releasing heavy metals. Examples of acceptable ranges of heavy metals from regulations in Germany, Switzerland and Austria can be found (Snellings et al., 2023) (Figure 2); however, the ranges seem to account for general applications in cement and concrete without any connection to specific secondary materials. When considering soil stabilization, another application of cementitious materials, the effects of a potential cement on soil environmental quality thresholds become a critical measure. Although reviewing regulations from across the globe, considerable differences can be seen (Figure 2) (Provoost et al., 2006).

Within these examples, either the amount of potential hazard within the source of contamination (e.g. total heavy metal content), or the threshold at which a potential receptor is deemed contaminated (e.g. soil quality) is considered. However, typically in contamination risk assessments, a source-pathway-receptor link is required for a contaminant to be deemed a hazard (Watts, 1998). As the primary pathway for heavy metals to a receptor is through solubilization into waterways, leaching tests provide critical information as to whether the use of a mineral-based waste-derived SCM presents a potential hazard risk within an application.

Leaching tests are typically percolation-based (CEN/TS 14405 and CEN/TC 351/TS-3) or batch-based (EN 12457-1, 2 or 3), in which a controlled amount of waste solid (S) is submerged in a controlled amount of liquid (L) solution (L/S ratio). The effect of solution pH, a key parameter for metal leaching, is also probed (CEN/TS 14429 and CEN/TS 14997). When considering national end-of-waste criteria for construction and demolition wastes, which also contains potential leachable metals, significant discrepancies in the testing methods and parameters and application categories between member states exist (Table 4) (Joint Research Centre et al., 2012). This further highlights the contrast that exists in this context when it is looked at through the lens of legislative bodies in different countries.

**Table 4.** Examples of testing approach and categorization for end-of-waste analysis for various European Member States (Austria, Czech Republic, Denmark, Finland, France, Italy and Sweden).

Member state	L/S	Categorization	Application	Leaching test
Austria	10 l/kg	Different grades	C&D in bound-unbound	EN 12457-4
Czech Republic	–	Classes	C&D in different applications based on classes	EN 12457-4
Denmark	L/S = 2 l/kg with a contact time of 24 hours	Categories	Residuals, C&D and polluted soil	EN 12457-1
Finland	L/S = 10 l/kg	Categories	C&D as well as ash from coal combustion	CEN/TS 14405 (basic characterization) and EN 12457-3 (compliance)
France	L/S = 10	Density function distribution	Alternative materials for road construction	NF EN 12457-2 or NF EN 12457-4 NF CEN/TS 14405
Italy	L/S = 10 l/kg on material <4 mm		Decree 22/979 the waste management-agricultural waste and C&D waste-ashes and slags	EN 12457-2
Sweden	L/S = 0.1 l/kg L/S = 10 l/kg	Free use and landfill cover	Recycling in construction- unbound form	CEN/TS 14405

C&D: construction and demolition; L/S: liquid–solid ratio.

Although these leaching tests primarily focused upon the granular, ‘unbound’ states, when used within both concrete or soil applications, SCMs will be ‘bound’ within cementitious and soil matrices. In this context, monolithic tests are more appropriate; however, there are currently no available harmonized standards on leaching tests for monolithic samples. A non-harmonized, technical specification, CEN/TS 16637-2 exists, which can provide a route to standardized values of monolithic leaching values.

To provide a possibility for the scientists and engineers in this area to better judge the hazardousness and potentials of the mineral-based waste products they are researching before diving deeper into available categorizations in national legislations, a figure has been created, summarizing, as far as the authors can reasonably find, limits (lower and higher limits) associated with national end-of-waste recycling criteria (Joint Research Centre et al., 2012) and soil environmental quality criteria (Figure 2) (Chen et al., 2018; Provoost et al., 2006). The thresholds are further compared with legislative limit values for total heavy metal contents for concrete in Germany, as well as leaching limit values regulating the use of secondary resources in bound applications in the Netherlands (Snellings et al., 2023). Soil environmental quality data in the United States is presented for comparison, highlighting significant deviations in regulatory limits (e.g. Cr, Cu, Zn), when compared to the other countries (Provoost et al., 2006). Although there are a number of limitations to this overview, it is clear that there are significant inconsistencies between the interpretation and execution of national end-of-waste threshold values. This presents a barrier to the widespread development of new waste-derived SCMs, and the use of waste materials in the construction sector as a whole.

When considering product-related regulations for environmental safety (REACH & CLP), within the cement industry, clinker is defined as a substance (chemical element and its compounds in the natural state) exempt from REACH regulation (Annex V) based on the grounds that the hazards/risks posed by cement clinker, after 180 years of worldwide manufacture, are so well known that it does not need to be registered with an agency (Taylor and Gibson, 2013). Cement is defined as a preparation (a mixture composed of two or more substances), which is also not subjected to REACH registration. This preparation includes the clinker substance and also further constituents (e.g. gypsum) which make up common cement mixture formulations.

A safety data sheet, with potential exposure scenarios, is required for cement and cement clinker, to ensure health and safety information is communicated to suppliers and customers. Furthermore, manufacturers are required to ensure that any inputted substances are registered, for example, fly ash, slag, silica fume and limestone. Once created, REACH dossiers are open to widespread commercial use. Therefore, associations often pool resources to ensure a full range of inputs for substances related to production.

As cement is a preparation, no C&L notification to ECHA is necessary. However, national bodies do require hazard information, in order to notify poison centres in case of emergency. To fulfil this obligation, a ‘standard formulas’ list was created for common cement preparations. This was created due to the difficulty in providing information for raw materials with highly variable (batch to batch) or unknown composition. Suppliers which comply with the standard formula lists are allowed to deviate from the obligations and requirements of the mixture compositions, as it is assumed that hazards do not change within the concentration ranges specified (CEMBUREAU – The European Cement Association, 2022; European Chemicals Agency, 2022).

Although clinker and common cement formulas benefit from a number of exemptions and pre-determined classifications, when considering the introduction of new waste-derived SCMs, a number of regulatory hurdles are evident. As new potential cements do not gain the attribute of historical use, a comprehensive physicochemical, toxicological and ecotoxicological assessment is required. This requires the development of a chemical safety assessment, which involves: collecting all potential hazards and thresholds for exposure considered as safe, measuring or estimating the dose or concentration to which humans or the environment may be exposed, and the ultimate risk characterization (European Chemicals Agency, 2023a, 2023b; European Committee for Standardization, 2017; Salvito et al., 2020). The complexity of this assessment is further increased when considering waste-derived SCMs are typically defined as substances of unknown or variable composition, which means that the number of constituents is relatively large, composition is variable and difficult to predict (European Chemicals Agency, 2010).

Although mineral-based waste-derived SCMs may meet end-of-waste criteria established at the national level, in order to access the European market as a whole, a REACH dossier is required. With waste management typically de-centralized, the costs of individual REACH assessments for each potential waste stream create a barrier to their widespread adoption. It is therefore critical that industry and researchers investigating waste-based materials pool their knowledge, increasing the weight of evidence towards the potential presence/or lack of presence of substance of concerns, therefore allowing for quick and accurate risk assessment, and lowering the barriers to REACH & CLP registration.

### Performance regulations for mineral-based waste use as SCMs

As noted in the previous section, the recycling transition is directed not only by environmental safety concerns (REACH & CLP) but also the technical performance (CPR, Eurocodes). When exploring the history of cement and CPR, you find that EN 197-1 (Cement – Part 1: Composition, specifications and conformity criteria for common cement) was the first harmonized standard in Europe (CEMBUREAU – The European Cement Association, 2020). Whilst the current version of the standard, EN 197-1:2011 has been in force, unamended for a number of years. In 2018, technical committee CEN/TC 51 (cement and building limes) published a draft amendment, prEN 197-1:2018. In 2021, technical report CEN/TR 16912:2021 outlined a dossier of technical information required for a new cement to achieve ‘fitness for intended use’ and the endorsement of the technical committee CEN/TC 51 for a new work item request to the European Commission (European Committee for Standardization, 2016). Further in 2021, EN 197-5:2021, a non-harmonized EU cement standard was published, which allows for clinker content reduction of up to 50%. Despite these reports, the lack of an updated *harmonized* technical documents, which allow for the *presumption of conformity*, has become a barrier to the widespread use and development of mineral-based waste-derived SCMs. With the proposed repeal of CPR presenting a more comprehensive essential requirement list, it is likely that further standard gaps will be identified, and an increasing barrier to their market access will become apparent (European Commission, 2022b).

It has been proposed that a future where performance-based standards, in combination with the digitalization of large datasets of cementitious mineral phases and their characteristics (rheology, strength, durability, toxicity), will facilitate a new wave of cement innovation (John et al., 2019; Li et al., 2022). In light of this shift, in a recently published draft of standard EN 197-6 ‘cement with recycled building materials’, a clear emphasis on performance testing has been made, allowing for the introduction of cements related to requirements based on exposure classes valid in the place of use. However, the most daunting challenge remains, as to how to design robust and *universally accepted* testing regimes, which translate to desired performance, when new cements, such as mineral-based waste derived SCMs, are placed in real world conditions (Juenger et al., 2011).

Moving to Eurocodes, in the upcoming update to the Eurocodes (generation 2), following mandate 515 from the European Commission, efforts have been made to allow for the introduction of ‘Green Concretes’. Eurocode 2 for structural concretes will allow for specimen testing at higher age (between 21 and 90 for a project) and also follows EN 206 (Concrete – Specification, performance, production and conformity) in introducing exposure classes, which will allow for new concrete compositions to be introduced following performance-based durability testing (Arrieta et al., 2023). Müller (2023) presented the ERC concept in detail as an example of a performance-based approach together with the latest development in European cement standards. It is key that a consensus is developed on determining the values critical for limit state analysis for structures containing mineral-based waste-derived SCMs so that engineers are confident in designing and specifying these materials within new structures. The further integration of non-destructive, in situ monitoring methods, in combination with wide-reaching databases, will further improve understanding and confidence in applying new materials, such as mineral-based waste-derived materials, in new structures and environments (Suchorzewski et al., 2023).

### Interfacing environmental safety and performance regulations for mineral-based waste use in the EU construction industry

**Figure 3.**
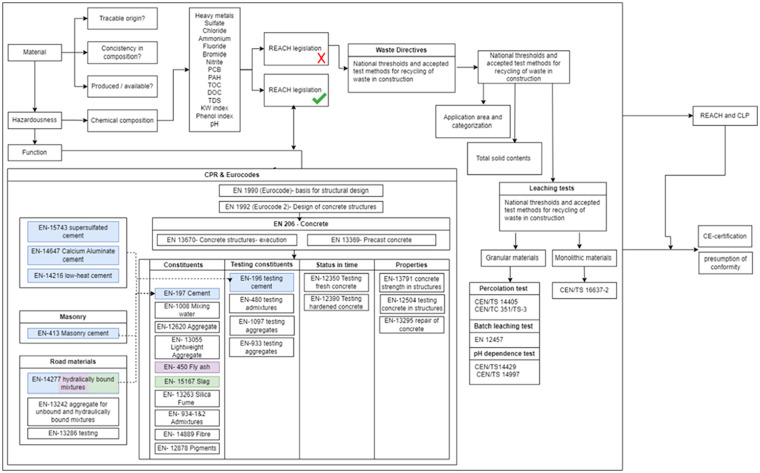
Schematic perspective on the major regulatory paths from production to application for mineral-based waste use in the European construction sector.

It clear that an integrated approach, through the Waste Framework Directive, REACH & CLP and harmonized standards: EN 197 (Cement), EN 206 (Concrete) and Eurocode 2 (Concrete Structures), is necessary for a construction project to benefit from the ‘presumption of conformity’ and the associated ease of market access for a proposed construction material. To provide a possibility for the scientists and engineers in this area to better plan the routes to take, to judge the potentials of the mineral-based waste products they are researching, before diving deeper into some specific area, a schematic perspective on the major regulatory paths (as far as the authors can find), from production to application view is presented (Figure 3). The figure highlights the importance of a multi-disciplinary approach when integrating mineral-based waste materials with a construction project and can serve as the foundation for future discussions (e.g. case studies) assessing the barriers for a particular mineral-based waste stream, within a particular application.

The value of a multi-disciplinary, multi-stakeholder approach has also been recognized by the German National Standardization Body, which has suggested a ‘concrete team’, which brings together all stakeholders, from material contractors to designers, to produce sustainable concrete formulations (European Committee for Standardization, 2013). A shift towards an integrated approach can also be seen with cement multi-nationals, whereby more companies are offering services that tailor cements for end-user specifications (Lehne and Preston, 2018).

## Concluding remarks: Towards a sustainable and circular construction sector

Within this manuscript, the authors aimed to explore and provide a perspective on the major regulatory hurdles for the widespread adoption of mineral-based waste materials, such as SCMs. A number of observations have been made. When considering the Waste Framework Directive, the emphasis on nationally defined end-of-waste criteria has created lack of clarity across Europe. The development of European harmonization would provide clarity and allow for the widespread use of mineral-derived waste in the construction industry. Within product-based environmental safety regulations, REACH & CLP, a historic lack of experience within the field and lack of mechanism to share information is creating a barrier to consensus development. It is key that platforms are developed to share expertise and lower the barriers to producing accurate and, where possible, common risk assessments. Within performance regulations, CPR, the lack of recent harmonized standards, presents a significant barrier to their use. With the shift towards performance-based standards, it is critical that a comprehensive and accepted testing regime is developed. When considering structural specifications, such as the Eurocodes, it is essential that consensus is developed regarding the key values for limit state analysis so that engineers are confident in designing and specifying recycled constituents within new structures.

Within this manuscript the current environmental safety and technical performance regulatory barriers to mineral waste use within the EU has been outlined, emphasizing the importance of a multi-stakeholder, multi-regulatory approach. The manuscript aims to establish a foundation for future discussions, which may expand the scope to consider further mineral waste-related legislation (e.g. demolition, excavation, life cycle assessment). Future work will also systematically explore, through quantitative analysis (e.g. time, resources), the potential of digitalization and automation, to catalyse existing consensus development procedures, helping to robustly determine the pathway towards achieving a material development timeline which is compatible with our climate obligations and societal needs. Furthermore, although the EU represents a major market and exporter of construction regulations, it critical that the regulatory barriers to mineral based waste use in other major jurisdictions (e.g. United States, United Kingdom and Australia) are assessed. Through building our understanding and overcoming the barriers associated with consensus-based procedures, we will catalyse the global integration waste materials into construction projects and facilitate the much-needed paradigm shift towards a sustainable and circular construction sector.
